# Peripheral dose measurements in cervical cancer radiotherapy: a comparison of volumetric modulated arc therapy and step-and-shoot IMRT techniques

**DOI:** 10.1186/1748-717X-9-61

**Published:** 2014-02-21

**Authors:** Ming X Jia, Xu Zhang, Ce Yin, Ge Feng, Na Li, Song Gao, Da W Liu

**Affiliations:** 1 Department of Radiation Oncology, Shengjing Hospital of China Medical University, Shenyang 110022, China; 2 Department of Gynecologic Oncology, Shengjing Hospital of China Medical University, Shenyang 110022, China; 3 Department of Radiation Oncology, The Fourth Hospital of China Medical University, Shenyang 110032, China

**Keywords:** Cervical cancer, Volumetric modulated arc therapy, Intensity modulated radiation therapy, Peripheral dose

## Abstract

**Purpose:**

The aim of this study was to investigate the peripheral doses resulting from volumetric modulated arc therapy (VMAT) and intensity modulated radiotherapy (IMRT) techniques in cervical cancer radiotherapy.

**Methods:**

Nine patients with cervical cancer had treatment planned with both VMAT and IMRT. A specially designed phantom was used for this study, with ion chambers placed at interest points approximating the position of the breast, thyroid, and lens. The peripheral doses at the phantom interest points were measured and compared between the VMAT and IMRT techniques.

**Results:**

VMAT provides a potential dosimetric advantage compared with IMRT. The mean (± standard deviation) peripheral dose to the breast point for 1 fraction (2 Gy) during VMAT measured 5.13 ± 0.96 mGy, compared with 9.04 ± 1.50 mGy for IMRT. At the thyroid and lens interest points, the mean (± standard deviation) peripheral dose during VMAT was 2.19 ± 0.33 and 2.16 ± 0.28 mGy, compared with 7.07 ± 0.76 and 6.97 ± 0.91 mGy for IMRT, respectively. VMAT reduced the monitor units used by 28% and shortened the treatment delivery time by 54% compared with IMRT.

**Conclusion:**

While the dosimetric results are similar for both techniques, VMAT results in a lower peripheral dose to the patient and reduces the monitor-unit usage and treatment delivery time compared with IMRT.

## Introduction

Radiotherapy is an important treatment modality for patients with cervical cancer. Over the last decade, interest in the use of intensity modulated radiotherapy (IMRT) to treat cervical cancer has been increasing. The IMRT technique has the potential benefit over conventional whole-pelvis irradiation of improving target dose coverage, reducing the volume of the organs at risk (OARs) that receive irradiation, and reducing the toxicity to normal tissue [1-4]. Despite the significant benefits of IMRT, there are some disadvantages. The technique usually requires multiple fixed-angle radiation beams, which can increase treatment delivery time. This has an impact on patient comfort, reproducibility of the treatment position, and intra-fraction motion. Moreover, IMRT uses a larger number of monitor units (MUs) compared with conventional conformal radiotherapy (CRT), leading to an increase in the amount of low-dose radiation received by the rest of the body. This raises the concern of secondary radiation-induced malignancy, which is of particular relevance to young patients or those with long future life expectancies [5-8].

A novel radiation technique, volumetric modulated arc therapy (VMAT), has recently been introduced in an attempt to overcome some of the limitations associated with fixed-field IMRT. VMAT allows continuous delivery of radiation by simultaneously varying the dose rate, the positions of the multileaf collimator, and the gantry rotation speed. Some studies demonstrate that VMAT can achieve highly conformal dose distributions, with improved target-volume coverage and sparing of normal tissues, compared with conventional radiotherapy techniques and IMRT [9-15]. VMAT also has the potential to offer additional advantages over conventional fixed-field IMRT in treatment-delivery efficiency, as a result of the reduction in both treatment delivery time and MU usage.

The peripheral dose is of clinical interest; it allows for estimation of the damage risk to those organs that are sensitive to relatively low doses of radiation. Such damage includes cataract formation in the lens of the eye and carcinogenesis, particularly in the thyroid and breast. The concept of the peripheral dose is normally associated with IMRT treatment, due to the increased number of MUs employed. Many researchers have investigated peripheral doses in IMRT [16-20].

The VMAT and IMRT techniques have been used to treat cervical cancer in clinical, yet to date, almost no data have been published that specifically address the peripheral dose generated by VMAT in patients with cervical cancer. The implementation of VMAT or IMRT in the treatment of cervical cancer raises concerns regarding the peripheral dose exposure. The purpose of this study was to investigate and compare the relative peripheral doses in VMAT and IMRT, planning both treatments in study patients and measuring peripheral doses in a specially designed phantom, using ion chambers approximating the position of the breast, thyroid, and lens tissue.

## Methods and materials

### Computed tomography simulation and planning objectives

Nine patients with cervical cancer, treated with the VMAT technique at our department between February 2013 and May 2013, were enrolled in this study. Ages ranged from 36 to 62 years, with a median age of 47 years. All of the patients had squamous cell carcinoma. Staging was performed according to the International Federation of Gynecology and Obstetrics (FIGO) classification. 5 patients had StageIIA and 4 patients had StageIIB cervical cancer. The studies was approved by Shengjing hospital of China Medical University's ethics committee.

All patients were immobilized in the supine position using the HipFix hip and pelvis immobilization system (CIVCO, Orange City, IA, USA). Computed tomography (CT) simulation was performed for each patient using the AQUILION 16-slice spiral CT scanner (Toshiba Medical Systems Corp., Otawara, Japan) with oral and intravenous contrast. Contiguous 3-mm slices were taken from the iliac crest to the ischial tuberosities. Each patient’s images were transported to the treatment planning system (Oncentra 4.1, Elekta AB, Stockholm, Sweden) to design the plan for VMAT. Each patient also had a plan designed for IMRT, using the same images. Two plans (VMAT and IMRT) were therefore designed for each patient, for a total of 18 treatment plans.

The gross tumor volume (GTV), clinical target volume (CTV), rectum, bladder, small bowel, and femoral heads were delineated in all patients by a single radiation oncologist. The GTV measured the volume of the primary tumor. The CTV comprised the GTV and any potential microscopic disease, including the entire uterus, parametrial tissues, the upper half of the vagina, and the regional lymph nodes in its calculation. The CTV was expanded uniformly by 8 mm in all directions to produce the planning target volume (PTV).

According to our treatment protocol, and referring to other studies [3,14,21], the dose prescription to the pelvis was set at 50 Gy, administered in 2 Gy fractions. Treatment plans were normalized to provide the mean dose to the PTV, in order to avoid any bias or rescaling effects in the comparison. The planning goal for both VMAT and IMRT was to obtain 95% of the prescribed dose to 98% of the PTV and not to exceed 105% as maximum dose. For the OARs including the rectum, bladder and small bowel, the dose received by 2% of the tissue volume (D_2%_) defined as the maximum dose was limit to 50 Gy. The complementary constraints (listed as V_40Gy_, the volume receiving 40 Gy of radiation) were < 40% for the rectum, < 50% for the bladder, < 25% for the small bowel, and < 5% for the femoral heads.

### IMRT and VMAT planning

In IMRT planning, 7 angles were used to evenly separate coplanar fields, and “step-and-shoot” delivery was used. A collapsed-cone convolution algorithm was used to calculate dosage, with a dose grid resolution of 3 mm. In VMAT planning, a collapsed-cone convolution algorithm was adopted, using 2 full arcs of a clockwise rotation from the initial angle of 182 degrees to the end angle of 178 degrees. For optimization between the calculation time and the accuracy of treatment planning, 4 degrees was set as the spacing unit. There were a total of 180 control points, with the collimator of 45 degrees. We used the Elekta Synergy linear accelerator (Elekta AB, Stockholm, Sweden) to implement both VMAT and IMRT plans. A high-energy 6-MV photon beam was used for both techniques.

### Dosimetric evaluation of VMAT and IMRT techniques

The quantitative plan evaluation was performed with a standard dose-volume histogram. For the PTV, the dose received by 98% of the tissue volume (D_98%_) was defined as the minimum dose, and the dose received by 2% of the tissue volume (D_2%_) was defined as the maximum dose. In addition, the volumes receiving at least 95% and at least 105% of the prescribed dose (V_95%_ and V_105%_) were reported. Treatment homogeneity was expressed as the difference between the doses covering 5% and 95% of the PTV (D_5%_ - D_95%_). The conformity index (CI_90%_) was defined as the ratio between the volume receiving at least 90% of the prescribed dose and the volume of the PTV. For the OARs, analysis included the mean dose, D_2%_ and V_40Gy_.

### Phantom and peripheral dose measurements

**Figure 1:**
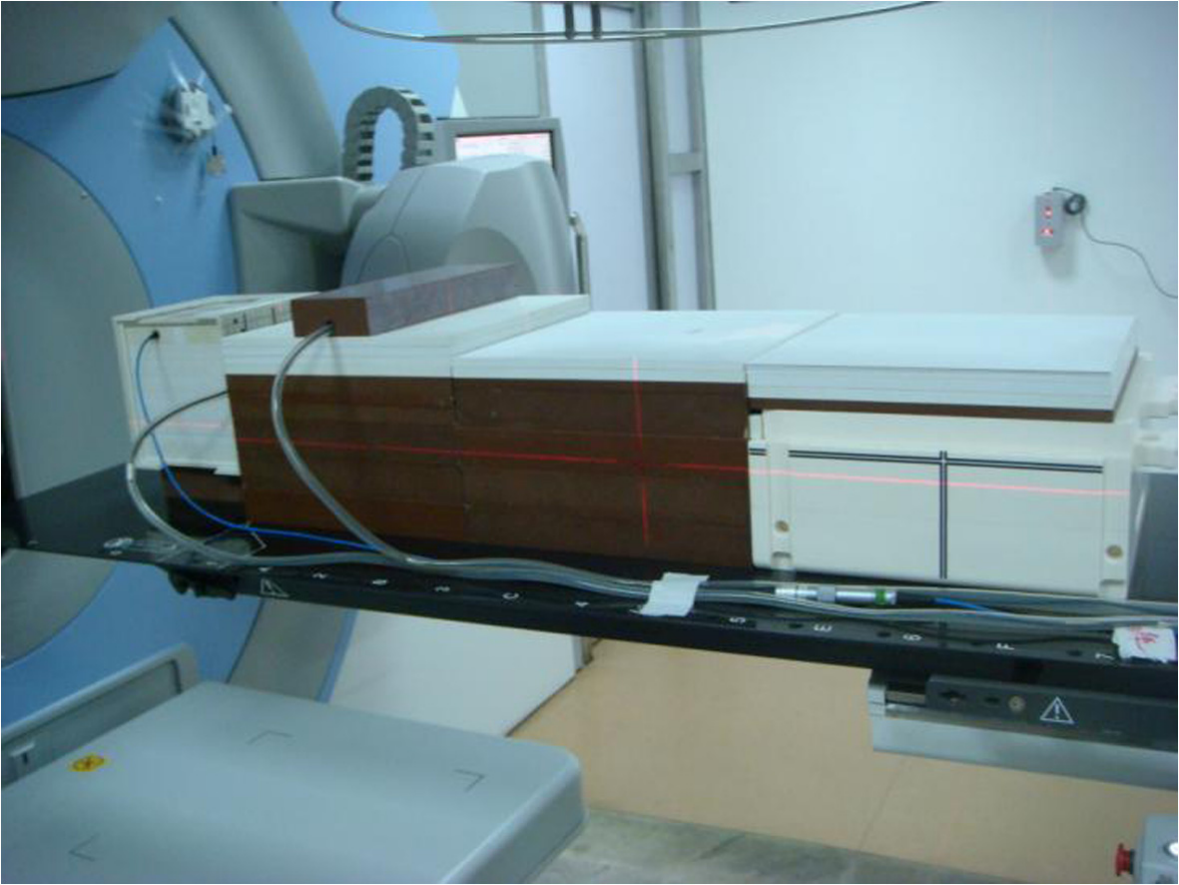
**Solid water slab phantom used to measure peripheral doses.** The positions of the 3 interest points are the breast, thyroid, and lens. The distance from the isocenter to the interest point is 35, 51, and 62 cm; the depth is 3, 2, and 1 cm, respectively.

An adult-sized phantom was assembled from solid water slabs (Figure 1) to simulate a cervical cancer radiotherapy patient. Using the average size of the 9 patients, the head of the phantom was created with a set of 20 × 20 cm^2^ rectangular solid water slabs, stacked to an anterior-posterior depth of 22 cm. The trunk of the phantom was created with 3 sets of 30 × 30 cm^2^ solid water slabs, lined up longitudinally to a length of 90 cm. One set of solid water slabs was stacked to a depth of 21 cm for the chest, with a slab of 4 cm thickness and 10 × 30 cm^2^ size used to represent breast tissue. Two sets of solid water slabs were stacked to a depth of 19 cm for the abdomen and pelvis. One slab of 4 cm thickness and 10 × 17 cm^2^ size was inserted between the head and the trunk to represent the neck. The positions of the measurement points in the phantom were determined according to the average positions of the breast, thyroid, and lens in the 9 patients. The breast position was set at a distance of 35 cm from the isocenter and a depth of 3 cm, the thyroid position was set at 51 cm from the isocenter and a depth of 2 cm, and the lens position was set at 62 cm from the isocenter and a depth of 0.5 cm.

The phantom was then used to simulate treatment. The phantom was positioned on the treatment couch so that the center of the “pelvis” coincided with the machine isocenter, and so that the longitudinal axis of the phantom was perpendicular to the plane of gantry rotation. The ion chambers (FC65-G, IBA Dosimetry GmbH, Schwarzenbruck, Germany) were placed within the phantom at the positions meant to represent the breast, thyroid, and lens. A single fraction of 2 Gy was then delivered to the isocenter, using either the VMAT or IMRT plan, as determined for each of the 9 patients; the peripheral doses at the 3 interest points were measured using the ion chambers. The number of MUs and the treatment delivery time were recorded separately for each plan. The treatment delivery time was calculated from beam-on to beam-off, including time for radiation delivery and gantry rotation. A total of 18 measurements were made (9 VMAT plans and 9 IMRT plans).

The peripheral dose was defined, for the purposes of this study, as the radiation dose anywhere outside the treatment field; it was composed of the scatter dose and the leakage dose. The leakage dose was defined as the dose at the more distant point (i.e., the lens dose), and the scatter dose was defined as the dose difference between the closest point outside the field edge and the most distant point (i.e., breast dose minus lens dose).

The ion chambers were calibrated by the dosimetry calibration laboratory of the National Institute of Standards, using a standard ^60^Co beam energy. The inherent uncertainty was ± 0.27% and the reproducibility was ± 0.13%. The total uncertainty associated with our dosimeter was estimated to be within 2.0%.

### Statistical analysis

The paired, 2-tailed Student’s t-test was used to compare the differences between the VMAT and IMRT techniques. Statistical analyses were conducted using Statistical Package for the Social Sciences (SPSS), version 12.0 (SPSS, Inc., Chicago, IL, USA). Statistical significance was assigned to *p*-values of < 0.05.

## Results

### Dosimetric comparison

Detailed dosimetric comparisons between the VMAT and IMRT plans are listed in Table 1. VMAT showed similar target dose coverage, with slightly improved homogeneity and conformality, compared with IMRT. VMAT had a lower maximum dose (D_2%_) in the PTV (*p* < 0.05). However, the test results indicated no significant difference in the homogeneity (D_5%_ - D_95%_) and conformity index (CI_90%_) in the PTV (*p* = 0.07; *p* = 0.06, respectively). The VMAT plan was better than the IMRT plan in sparing the small bowel; the average mean dose to the small bowel was reduced by 4.5% with VMAT (*p* < 0.05). There were no significant differences in other dosimetric parameters (mean dose, D_2%_ and V_40Gy_ ) at the other OARs (rectum, bladder, and femoral heads) (*p* > 0.05).

**Table 1:** Dosimetric comparison between VMAT and IMRT plans: planned target volume and organs at risk

		**VMAT**	**IMRT**	***p*-value**
PTV				
	D_2%_ (Gy)	51.9 ± 0.5	52.2 ± 0.3	0.02
	D_98%_ (Gy)	47.4 ± 1.6	48.0 ± 0.3	0.36
	V_95%_ (%)	98.7 ± 0.8	98.7 ± 0.4	0.59
	V_105%_ (%)	0.5 ± 0.4	1.3 ± 1.1	0.22
	D_5%_ - D_95%_ (Gy)	3.3 ± 1.1	3.0 ± 0.3	0.07
	CI_90_	1.5 ± 0.3	1.7 ± 0.2	0.06
Rectum				
	Mean (Gy)	37.1 ± 1.5	38.1 ± 1.7	0.21
	D_2%_ (Gy)	49.5 ± 0.6	49.4 ± 0.9	0.60
	V_40Gy_ (%)	39.2 ± 5.1	40.7 ± 5.1	0.51
Bladder				
	Mean (Gy)	38.1 ± 1.4	38.8 ± 1.9	0.22
	D_2%_ (Gy)	50.4 ± 0.9	50.3 ± 0.5	0.77
	V_40Gy_ (%)	47.3 ± 4.0	46.6 ± 5.6	0.63
Small bowel				
	Mean (Gy)	28.8 ± 2.5	30.1 ± 1.4	0.03
	D_2%_ (Gy)	49.5 ± 0.8	49.9 ± 0.5	0.29
	V_40Gy_ (%)	20.3 ± 3.3	21.3 ± 3.5	0.41
Left femoral head				
	D_2%_ (Gy)	39.8 ± 1.7	40.3 ± 4.1	0.13
	V_40Gy_ (%)	2.3 ± 1.4	2.9 ± 1.9	0.46
Right femoral head				
	D_2%_ (Gy)	39.1 ± 3.5	39.2 ± 5.0	0.18
	V_40Gy_ (%)	2.5 ± 1.7	2.6 ± 1.9	0.60

PTV: planning target volume; VMAT: volumetric modulated arc therapy; IMRT: intensity-modulated radiotherapy; D_2%_: dose received by 2% of the tissue; D_98%_: dose received by 98% of the tissue; V_95%_: the volume receiving at least 95% of the prescribed dose; V_105%_: the volume receiving at least 105% of the prescribed dose; D_5%_ - D_95%:_ difference between the dose covering 5% and 95% of the planning target volume; CI_90%_: conformity index, defined as the ratio between the volume receiving at least 90% of the prescribed dose and the planning target volume; V_40Gy:_ volume receiving a dose of 40 Gy.

### Peripheral dose comparison

Table 2 shows the measured peripheral dose at the breast, thyroid, and lens for both VMAT and IMRT. The doses at the 3 interest points were lower for VMAT than for IMRT. The mean (± standard deviation) dose to the breast point for 1 fraction (2 Gy) of VMAT measured 5.13 ± 0.96 mGy, compared with 9.04 ± 1.50 mGy for IMRT. This resulted in a VMAT/IMRT mean breast dose ratio of 0.57. For the thyroid interest point, the mean dose from VMAT was 2.19 ± 0.33 mGy, and from IMRT was 7.07 ± 0.76 mGy, giving a mean thyroid dose ratio of 0.31. For the lens interest point, the mean dose from VMAT was 2.16 ± 0.28 mGy, and from IMRT was 6.97 ± 0.91 mGy, giving a mean lens dose ratio of 0.31. All 3 interest points displayed significant differences in the peripheral doses between VMAT and IMRT (*p* < 0.05).

**Table 2:** Comparison of peripheral dose, monitor units, and treatment delivery time

	**VMAT**	**IMRT**	***p*-value**
Breast (mGy)	5.13 ± 0.96	9.04 ± 1.50	<0.01
Thyroid (mGy)	2.19 ± 0.33	7.07 ± 0.76	<0.01
Lens (mGy)	2.16 ± 0.28	6.97 ± 0.91	<0.01
MUs	784.9 ± 179.2	1086.1 ± 184.6	0.01
Time (min)	6.12 ± 1.51	13.15 ± 0.83	<0.01

VMAT: volumetric modulated arc therapy; IMRT: intensity-modulated radiotherapy.

In order to estimate the total dose delivered over an entire treatment course, the single fraction mean values were multiplied by 25. For the VMAT technique, the calculated total dose to the breast, thyroid, and lens points was 128, 55, and 54 mGy, respectively. These were each lower than the calculated total dose obtained with IMRT, of 226, 177, and 174 mGy, respectively. The calculated scatter dose to the breast from VMAT and IMRT was approximately 74 and 52 mGy, respectively. The scatter dose was slightly higher for VMAT than for IMRT, but the magnitude of the dose was quite small. The leakage dose from VMAT and IMRT was about 54 and 174 mGy, respectively; the leakage dose was much lower for VMAT than for IMRT.

### MU and treatment delivery time comparison

The MUs and treatment delivery time associated with VMAT and IMRT are also shown in Table 2. Both measurements were lower for VMAT than for IMRT. Compared with IMRT, which used 1086 ± 185 MUs, VMAT used only 785 ± 179 MUs, a reduction of 28%. The treatment delivery time in VMAT (6.12 min) was reduced by 54% compared with IMRT (13.15 min). These differences were statistically significant (*p* < 0.05).

## Discussion

The results of the present study indicate that, although VMAT and IMRT have similar target dose coverage, VMAT has improved homogeneity and conformality, and is able to treat cervical cancer more efficiently with less damage to the small bowel. Cozzi et al. [14] conducted a treatment planning study comparing volumetric arc modulation and fixed-field IMRT for cervix uteri radiotherapy. Their study showed that the treatments provide the same coverage but arc therapy offers better homogeneity and conformality to the PTV. Arc therapy also reduces the mean dose to the bladder and rectum, which ranges from about 4 to 6 Gy. Cozzi et al’s results are slightly better than ours in sparing the bladder and rectum, presumably because they have extensive experience with VMAT planning. However, a discussion of the dosimetric advantage of VMAT is not the focal point of our study; we have focused on the peripheral dose resulting from VMAT in cervical cancer radiotherapy.

The peripheral dose arises primarily from scatter, generated by the patient or phantom, the machine collimators, and the walls of the room, and from leakage through the machine’s shielding and collimators. Many studies have reported the peripheral doses resulting from various radiotherapy techniques [19,20,22-26]. Kase et al. [22] measured the peripheral dose from secondary radiation outside an open treatment field. They found that the peripheral dose can be described by a simple exponential function of distance from the central axis of the radiation field for all energies and field sizes. The peripheral dose depends on the machine collimators, the field size, and the distance from the field. Machine collimator scatter contributes between 20% and 40% of the dose outside the treatment field; leakage radiation contributes very little.

Sharma et al. [23] reported the peripheral dose resulting from a uniform dynamic multileaf collimation (DMLC) field. They measured the different doses in a phantom that compared static open fields to those using moving strip fields of varying width, in an attempt to simulate DMLC delivery. Their study showed that all dynamic fields deliver a higher relative peripheral dose compared with the corresponding static open fields. However, peripheral doses close to the field edge can be higher for static fields than for certain DMLC comparison fields. They also carried out a feasibility study that used peripheral dose data from uniform DMLC fields to estimate out-of-field organ doses from IMRT treatment [20].

Mansur et al. [19] compared peripheral doses between IMRT and 3-dimensional CRT techniques in pediatric radiotherapy. Their study showed that IMRT delivery results in lower peripheral doses at points near the target, presumably due to reduced internal scatter from the smaller effective field size used in sliding window DMLC. Conversely, IMRT delivery results in higher doses to more distant points, presumably due to the higher MUs involved and the resulting increased head leakage. Since the magnitude of the doses at the distant points is much smaller than that seen at the closer points, the overall absolute peripheral dose is similar for both techniques.

Some researchers have pointed out that a higher peripheral dose may occur with the IMRT technique [24,25]; others have reported a similar peripheral dose for IMRT and 3-dimensional CRT, at least in selected cases [26]; still others have reported a lower peripheral dose for IMRT in some cases [27]. These discrepancies may result from the fact that VMAT is a novel radiation therapy technique. It is important to investigate the peripheral dose and the OAR dose outside the treatment field in cervical cancer radiotherapy using VMAT and to compare these results with IMRT. A little data have been reported on the peripheral doses resulting from VMAT in cervical cancer radiotherapy. Qiu Y et al. described the peripheral doses for gynecological patients undergoing IMRT or RapidArc (Varian Medical Systems, Palo Alto, CA) with kilovoltage cone beam CT [28]. This study shows that the IMRT leakage dose is approximately 6 cGy, uniformly distributed throughout the patient’s body, while the leakage dose from RapidArc is about 3 cGy, using a prescribed dose of 45 Gy. Scatter doses from IMRT and RapidArc are similar in magnitude, reportedly about 5 cGy at the breast. Cozzi et al. also carried out a study in cervix uteri radiotherapy showing that RapidArc had a better profile than IMRT concerning peripheral doses, and RapidArc can reduce the peripheral dose from about 8% at 5 cm to about 30% at 15 cm from the PTV surface compared with IMRT. This result is in full support to our measurements.

Our phantom measurements reveal that the peripheral dose is always lower for VMAT than for IMRT, decreasing with increasing distance of measurement for both techniques. The VMAT dose at points closer to the beam edge (i.e., the breast point) is lower than the IMRT dose by about half, and the dose at more distant points (i.e., thyroid and lens points) is about a third lower. These differences can be explained by considering the 2 components of the phantom’s peripheral dose: internal scatter and accelerator head leakage. Internal scatter primarily affects tissues or organs at points closer to the beam edge and is directly proportional to the field size. Accelerator head leakage affects the entire body equally and is directly proportional to the MUs employed.

In the VMAT technique, continuous delivery is achieved by simultaneously varying the dose rate, the positions of the multileaf collimator, and the gantry rotation speed. Compared with IMRT, VMAT reduces MU usage and treatment delivery time, and the modulated beam has a relatively large field size. This means that the leakage contribution to the peripheral dose becomes quite small and the peripheral dose at distant points is lower. While the scatter contribution to the peripheral dose becomes larger, the peripheral dose at points closer to the beam edge increases. The leakage dose and scatter dose contribute approximately 42% and 58%, respectively, of the total peripheral dose from VMAT.

With the IMRT technique, “step-and-shoot” delivery is administered at different gantry angles. Compared with VMAT, IMRT increases both the MU usage and treatment delivery time, and the modulated beam has a relatively small field size. This means that the leakage contribution to the peripheral dose becomes quite large and the peripheral dose at distant points is higher. While the scatter contribution to the peripheral dose becomes relatively small, the peripheral dose at points closer to the beam edge decreases. The leakage dose and scatter dose contribute approximately 77% and 23%, respectively, of the total peripheral dose from IMRT.

Our study found that the scatter dose predominates in the peripheral dose in VMAT, while the leakage dose predominates in IMRT. Our scatter doses to the breast from both VMAT and IMRT are similar to those reported by Qiu et al. [28], but our leakage doses are larger. The difference possibly results from the use of different study methods and prescribed doses.

Knowledge of the peripheral dose resulting from VMAT and IMRT techniques in cervical cancer radiotherapy is important and of clinical interest. Physicians must be able to estimate the dose to the non-treated organs and the resultant risk to patients. An understanding of the peripheral dose allows a more complete assessment of the radiation dose to long-term survivors.

## Conclusion

The VMAT technique is able to treat cervical cancer more efficiently than the IMRT technique. VMAT provides a potential dosimetric advantage, results in a lower peripheral dose to the patient, and reduces the MU usage and treatment delivery time.

## **Consent**

Written informed consent was obtained from the patient for the publication of this report and any accompanying images.

## Competing interests

The authors declare that they have no competing interests.

## Authors’ contributions

MX Jia carried out the studies about peripheral dose for cervical cancer, put forward the whole idea and drafted the manuscript. X Zhang, C Yin and G Feng participated in the measurement. N Li participated in the statistical analysis of the data. S Gao involved in delineating the GTV, CTV and OARs. DW Liu participated in the phantom mimicking. All authors read and approved the final manuscript.
